# Transcatheter aortic valve implantation for aortic regurgitation in HeartMate II supported patient using Myval THV: a case report

**DOI:** 10.1093/omcr/omad086

**Published:** 2023-10-23

**Authors:** Mohamad Hamieh, Zahra Nassereddine, Malek Moussa, Firas Al Ali, Mohamad Dbouk, Mohamad Saab

**Affiliations:** Department of Cardiology, Beirut Cardiac Institute, Beirut, Lebanon; Department of Cardiology, Beirut Cardiac Institute, Beirut, Lebanon; Department of Cardiology, Beirut Cardiac Institute, Beirut, Lebanon; Department of Cardiovascular Surgery, Beirut Cardiac Institute, Beirut, Lebanon; Department of Cardiology, Beirut Cardiac Institute, Beirut, Lebanon; Department of Cardiovascular Surgery, Beirut Cardiac Institute, Beirut, Lebanon

## Abstract

De novo aortic regurgitation (AR) presents a great challenge following left ventricular assist device (LVAD) implantation and requires valve replacement in some cases. Patients with LVAD are frequently those who underwent multiple previous sternotomies or suffer from multiple comorbidities. Thus, they are at high surgical risk for further sternotomy. Transcatheter aortic valve implantation (TAVI) previously approved for treatment of severe aortic stenosis is also used for this category of patients. Here, we report the case of a young female patient supported with heart mate II LVAD who presented with severe de novo AR. The patient was successfully treated with TAVI using Myval trancatheter heart valve (THV) in our center. To our knowledge, our patient is the first to be treated with such type of valve using TAVI procedure in LVAD supported patients.

## INTRODUCTION

The number of patients supported with left ventricular assist device (LVAD) is growing worldwide as destination therapy, bridge to decision, or bridge to transplantation after the results of REMATCH trial [1]. The development of aortic regurgitation (AR) remains an important cause of recurrent heart failure after LVAD implantation. It occurs in 25% of patients in both pulsatile and continuous flow devices [1]. Significant AR is mostly seen in continuous LVAD HeartMate II [2–4]. The pathophysiological mechanisms include changes in aortic valve velocity and luminal pressure as well as changes of aortic valve leaflets leading to cusps remodeling and fusion [2, 4, 5]. Multiple associated risk factors were described including long support duration, continuous-flow pumps, advanced age, low body surface area, systemic hypertension and continuously closed aortic valve. The management of mild to moderate AR and asymptomatic severe AR is based on medical therapy, pump speed optimization and close follow-up. Intervention is reserved for those who have significant AR associated with clinical heart failure [5, 6]. Heart transplantation or surgical aortic valve replacement if feasible are the best options. Transcatheter aortic valve implantation (TAVI) is considered an alternative option in high-risk surgical patients [6].

## CASE PRESENTATION

**Figure 1:**
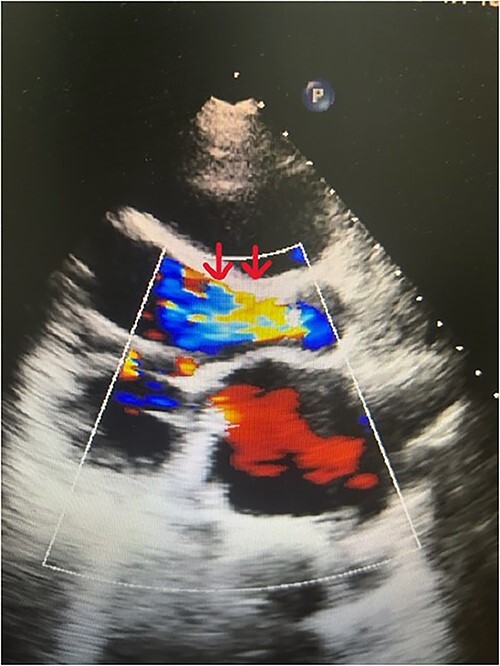
Doppler echocardiography shows severe AR (red arrows).

**Figure 2:**
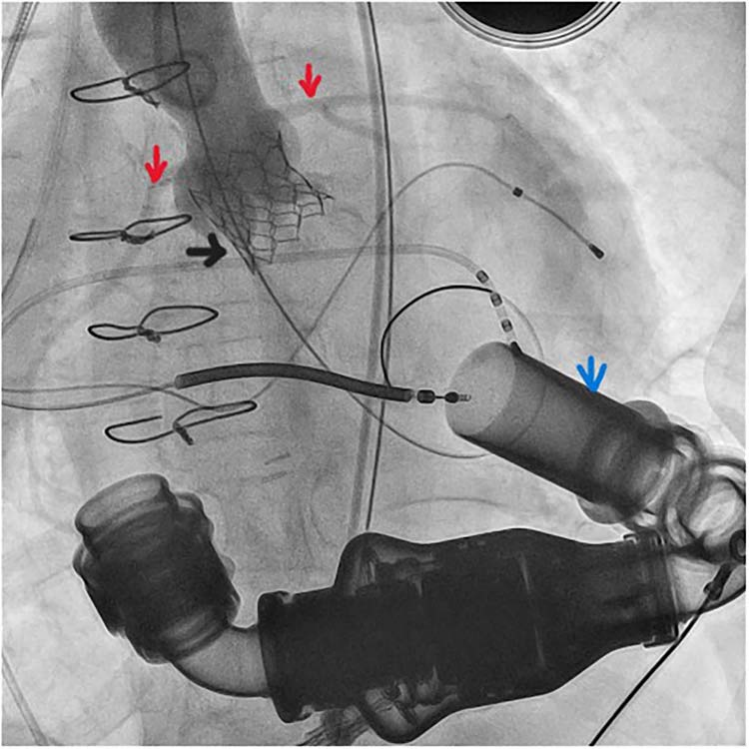
Aortography following valve deployment (black arrow), note the LVAD inflow canula (blue arrow) and the patent coronaries (red arrows).

We present the case of a 41-year-old female with a previous history of dilated cardiomyopathy and no other relevant medical conditions otherwise. She underwent mitral and tricuspid valve repair for severe mitral and tricuspid regurgitation respectively 11 years ago. One year later, she was supported with a HEARTMATE II (LVAS; Thoratec Corporation, Pleasanton, CA, USA) device for worsening heart failure not responsive to medical therapy. Heart transplantation was not feasible due to shortage of organ donation in our country. Three years later, she underwent a third sternotomy for pump exchange due to pump failure and received another HEARTMATE II pump (see timeline). Following the last surgery, only minimal AR was noted. Few years later, the patient started to have symptoms of dyspnea and fatigue and she had multiple hospital admissions for recurrent pulmonary edema. Transthoracic echocardiogram (TTE) showed severe AR with dilated left ventricle without change in device speed (Fig. 1). The optimization of the medical treatment and LVAD speed failed to relieve her symptoms. Therefore, she was again mentioned at the top of the waiting list for heart transplantation. Due to shortage of organ donation, the high surgical risk and the clinical urgency, TAVI procedure is considered after multidisciplinary discussion. Under a conscious sedation, the left femoral artery and the left femoral vein were obtained with a 6Fr and 7Fr introducer, respectively. Then, a surgical dissection of the right femoral artery was done followed by insertion of a 14Fr sheath. Under fluoroscopy, aortic angiography showed the severe AR. After reducing the pump speed and rapid ventricular pacing at a rate of 180 bpm, an intermediate size Myval™ trancatheter heart valve (THV) (Meril Life Sciences Pvt. Ltd., Vapi, Gujarat, India) was deployed successfully. The size of the implanted THV was 24.5 mm slightly larger than the actual annulus diameter measured on pre procedural computed tomography. The aortic angiography showed no residual regurgitation or paravalvular leak (Fig. 2). No significant transprosthetic pressure gradient was noted.

**Table:** Timeline:

2010	2011	2012	2015	2022
Diagnosis of cardiomyopathy	Mitral and tricuspid surgery	LVAD implantation	Pump exchange	TAVI for AR

**Figure 3:**
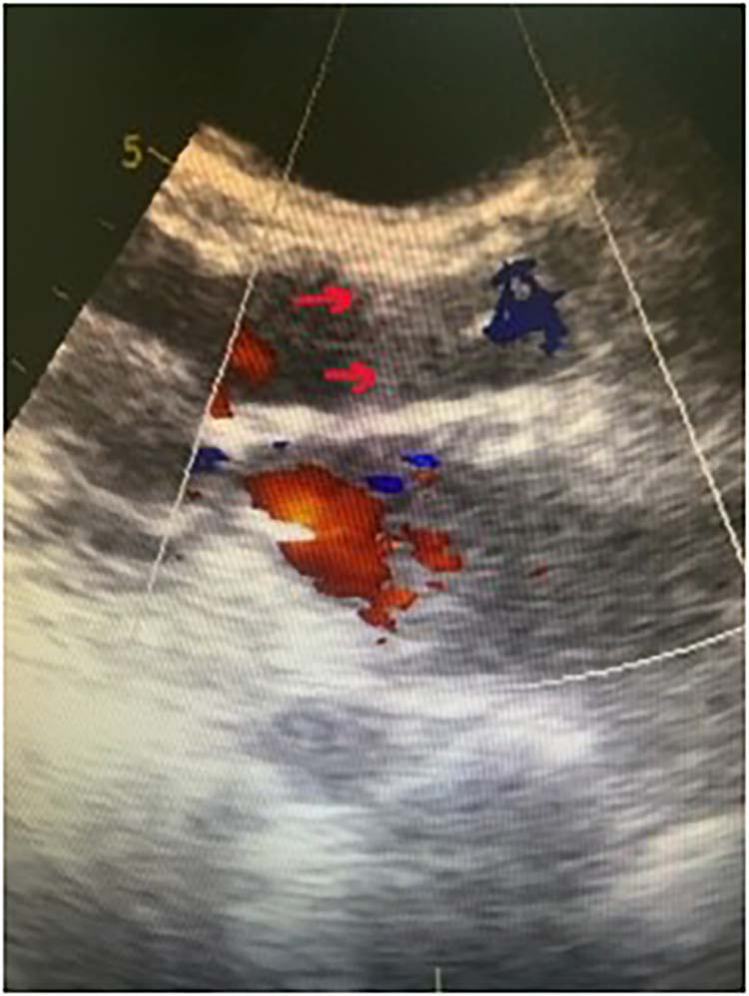
Doppler echocardiography after TAVI procedure shows valve in place (red arrows) and no residual regurgitation.

Following the procedure, the patient noted a marked clinical improvement. TTE showed a permanently closed aortic bioprosthesis with no valvular regurgitation or paravalvular leak (Fig. 3). Three days later the patient had repetitive non sustained ventricular tachycardia, TTE revealed a suction event managed with decreasing the pump speed. Furthermore, we noted an increase in blood pressure after TAVI procedure treated with candesartan. The patient was discharged home 1 week later in stable condition. TTE done 1 year later showed good function of the aortic prosthesis with no regurgitation.

## DISCUSSION

Considering TAVI for pure AR is not widely used because of lack of anchoring calcification, dilated aortic root and the risk of migration. Off label use of TAVI in pure AR was considered in many patients with high surgical risk as well as in patients with LVAD [5]. The first successful TAVI procedure intended to treat severe AR in an unstable patient supported with HeartMate II LVAD was first published in 2011 [7]. Thereafter, multiple THV were used in this purpose with different success rate, mainly Medtronic CoreValve, Evolut and Sapien 3 [8–9]. They conclude that better outcomes were associated with new generation THV. Furthermore, the use of self-expandable THV were preferred over balloon-expandable THV in patients with pure AR either in LVAD supported patients or non LVAD patients [10]. Dhillon and colleagues, recently reported a case series of four consecutives patients supported with LVAD treated successfully with self-expandable THV (Medtronic), only one patient required rescue intervention with a second valve [10].

To our knowledge, this is the first case of severe AR treated using Myval THV in LVAD HeartMate II patients. A single other case of a patient with HeartMate III was treated for severe AR with same valve in Italy 2021. They used an oversized Myval THV (32 mm) with good final results [11].

In our patient, we decided to use this particular valve because of the availability of larger sizes which allowed tight anchoring to aortic root, our center experience with it and its lower cost compared to other valve systems. Myval™ THV is a European Conformity-marked newer-generation balloon-expandable THV characterized by a nickel-cobalt alloy frame composed of a single element- hexagon arranged in a hybrid honeycomb fashion. This valve appears at least equal to other THV commonly used in setting of de novo AR following LVAD.

In the absence of dedicated THV approved for pure native AR, the success rate of the intervention mostly depends upon the experience of the center with the valve type.

## CONCLUSION

In our case, the use of our usual THV Myval in the treatment of AR in a patient supported with LVAD appeared safe and effective. The prosthesis remained in place one year following the procedure without valvular regurgitation. New dedicated prostheses for pure AR are under investigations and they are promising in patients with LVAD. Long term outcomes following TAVI in LVAD supported patients need to be determined.

## Data Availability

Data are available on request from the corresponding authors.
